# ZLM-7 Blocks Breast Cancer Progression by Inhibiting MDM2 via Upregulation of 14-3-3 Sigma

**DOI:** 10.3390/ph15070874

**Published:** 2022-07-15

**Authors:** Min Wen, Zi-Zheng Zou, Tiao Luo, Xuan Li, Su-You Liu, Ji-Jia Li, Zhi-Yong Luo

**Affiliations:** 1 Department of Biochemistry and Molecular Biology, Hunan Province Key Laboratory of Basic and Applied Hematology, Hunan Key Laboratory of Animal Models for Human Diseases, School of Life Sciences, Xiangya School of Medicine, Central South University, Changsha 410008, China; xixiwmtaohua@csu.edu.cn (M.W.); zouzizheng@csu.edu.cn (Z.-Z.Z.); 202501026@csu.edu.cn (X.L.); 2 Department of Biochemistry and Molecular Biology, Yiyang Medical College, Yiyang 413000, China; 3 Hunan Key Laboratory of Oral Health Research, Xiangya Stomatological Hospital, Xiangya School of Stomatology, Central South University, Changsha 410008, China; luotiao@csu.edu.cn; 4 Department of Medicinal Chemistry, School of Pharmaceutical Sciences, Central South University, Changsha 410006, China; suyouliu@csu.edu.cn; 5 Center of Stomatology, Xiangya Hospital, Central South University, Changsha 410008, China; 6 Research Center of Oral and Maxillofacial Tumor, Xiangya Hospital, Central South University, Changsha 410008, China; 7 Institute of Oral Cancer and Precancerous Lesions, Central South University, Changsha 410008, China

**Keywords:** breast cancer, ZLM-7, 14-3-3 sigma, MDM2, cell cycle

## Abstract

Breast cancer is one of the most prevalent malignancies with poor prognosis. Inhibition of angiogenesis is becoming a valid and evident therapeutic strategy to treat cancer. Recent studies uncovered the antiangiogenic activity of ZLM-7 (a combretastain A-4 derivative), but the regulatory mechanism is unclear. ZLM-7 treatment was applied in estrogen receptor-positive cell MCF-7, triple-negative breast cancer cell MDA-MB-231 and xenograft models. Transfections were conducted to overexpress or knockdown targeted genes. The gene and protein expressions were measured by qPCR and Western blotting assay, respectively. Cell proliferation and apoptosis were evaluated using the CCK8 method, clone formation assay and flow cytometry. We found that ZLM-7 upregulated 14-3-3 sigma expression but downregulated MDM2 expression in breast cancer cells. ZLM-7 delayed cell proliferation, promoted apoptosis and blocked cell-cycle progression in human breast cancer cells in vitro, while those effects were abolished by 14-3-3 sigma knockdown; overexpression of 14-3-3 sigma reproduced the actions of ZLM-7 on the cell cycle, which could be reversed by MDM2 overexpression. In xenograft models, ZLM-7 treatment significantly inhibited tumor growth while the inhibition was attenuated when 14-3-3 sigma was silenced. Collectively, ZLM-7 could inhibit MDM2 via upregulating 14-3-3 sigma expression, thereby blocking the breast cancer progression.

## 1. Introduction

Breast cancer, which originates in mammary epithelial cells, is one of the foremost prevalent cancers in females worldwide. According to the data from Global Cancer Statistics 2020, a striking number of over two million new breast cancer cases are identified per year, accounting for 6.9% of overall cancer deaths. If diagnosed with metastatic diseases, the overall 5-year survival rate of breast cancer sufferers was only 30% [1]. Although advances in diagnostics and therapeutic modalities had been made in recent years, the prognosis of advanced breast cancer is still undesirable owning to high recurrence and metastasis rate, early diagnosis failure, as well as toxicity and resistance associated with drugs [2,3]. Therefore, further investigation of molecular mechanisms of breast oncogenesis is warranted for the development of novel therapeutic strategies to achieve better outcomes.

Combretastatin A-4 (CA-4), which is a natural product obtained from Combretum caffrum, is one of the most effective microtubule-targeting inhibitors (MTAs) that display both antimitotic and antiangiogenic properties against tumors [4]. CA-4 had been previously proposed to be a potential agent for the treatment of breast cancer [5]. In addition, CA-4 could inhibit microtubule polymerization, cell migration and tumor growth in bladder cancer, further suggesting the antitumor activities of CA-4 [6]. Recently, a novel CA-4 analog TP5 was also reported to display potent antitumor activities in inhibiting tubulin polymerization, suppressing cell migration, arresting cells at the G2/M phase, and subsequently inducing apoptosis [7]. However, it was revealed that CA-4 could easily isomerize from the less stable cis-isomer to trans-isomer to achieve a more stable state, which leads to a substantial decline in its antitubulin and cytotoxic potency [8]. Thus, a number of cis-restricted analogs of CA-4 have been designed and synthesized to overcome this drawback [9]. ZLM-7 is another cis-restricted sulfide derivative of CA-4, and had been previously demonstrated to exhibit comparable antiproliferative and antitubulin activities to the activities of CA-4, while the toxicity of ZLM-7 turned out to be significantly lower [10]. Moreover, several studies also revealed the antiangiogenic action of ZLM-7 in breast cancer by blocking the vascular endothelial growth factor (VEGF) singling in xenograft mouse models [10,11], suggesting that ZLM-7 could be a promising compound for the inhibition of breast carcinoma.

Accumulating evidence suggests that the 14-3-3 protein family is an active participant in the regulation of cell-cycle progression and associated signal transduction by interacting with various protein ligands, such as kinases, phosphatases and transmembrane receptors [12]. Therefore, 14-3-3 sigma could be an important regulator in oncogenesis. Indeed, numerous studies have uncovered the intimacy of 14-3-3 sigma with tumor growth and progression, although the exact molecular mechanisms of 14-3-3 sigma are not completely understood [13]. Moreover, 14-3-3 sigma is traditionally proposed to be a tumor suppressor by interacting with the tumor protein p53 and other antioncogenes [14]. Several studies have also suggested that 14-3-3 sigma could facilitate tumor invasion and metastasis via specific mechanisms in various cancers [15,16,17]. In breast cancer, decreased expression of 14-3-3 sigma was found to contribute to the neoplastic transformation of epithelial cells by proteomic analysis [18]; meanwhile, it was also reported that overexpressed 14-3-3 sigma could delay the cell-cycle progression of breast cancer cells from entering the S phase by blocking the activity of cell-cycle regulatory proteins, such as cyclin-dependent kinases (CDKs) [19]. Those findings implicate the regulatory role of 14-3-3 sigma in breast cancers. We previously found that treatment of ZLM-7 could upregulate the expression of 14-3-3 sigma in breast cancer cell lines (data unpublished), prompting us to explore the action of ZLM-7 in the regulation of the cell cycle via 14-3-3 sigma in the present study.

Apart from ZLM-7, 2-Methoxy-5 ((3,4,5-trimethosyphenyl) seleninyl) phenol (SQ) is another synthesized structural analogue of CA-4 with a proven cell-cycle blocking effect that has been investigated to be a potential treatment for breast cancer. It has been reported that SQ exhibited a superior anticancer effect than CA-4 via inhibiting mouse double minute 2 (MDM2) and inducing cell-cycle arrest and subsequent apoptosis [20,21]. Being a pivotal negative mediator of the tumor suppressor p53, the MDM2 protein was able to suppress the transcriptional activity of p53 and thus has been an attractive target for cancer treatment by inhibiting its function [22]. Overexpressed MDM2 in tumors has been shown to correlate to a poor prognosis, especially in patients diagnosed with breast cancers [23]. Interestingly, 14-3-3 sigma could inhibit MDM2 function and therefore block MDM2-mediated p53 ubiquitination and nuclear export to suppress tumorigenesis [14,24]. Those observations further emphasize the regulatory function of 14-3-3 sigma in the p53-MDM2 pathway in cancers.

The objective of this study was to explore the potential regulatory machinery of ZLM-7 on the 14-3-3 sigma/MDM2 signal axis and to provide new insights for the therapeutic targets of breast cancer.

## 2. Materials and Methods

### 2.1. Cell Culture and Treatment

For the purposes of this study, we chose two human breast cancer cell lines, MCF-7 and MDA-MB-231, and a mammary epithelial cell line, MCF-10A. All cell lines were originally obtained from the American Type Culture Collection (ATCC, Manassas, VA, USA) and were cultured in DMEM (Gibco, Grand island, NY, USA) supplemented with 10% fetal bovine serum and 100 U/mL penicillin and streptomycin in a humidified incubator at 37 °C with 5% CO_2_. ZLM-7 was synthesized by research group of professor Liu Suyou, School of Pharmacy, Xiangya Medical College, Central South University (Patent No: CN1040039). ZLM-7 treatment was achieved by treating the cells with ZLM-7 at a concentration of 10 nM for 8 h prior to subsequent experiments [11].

### 2.2. Cell Transfection

The pcDNA3.1 vectors expressing 14-3-3 sigma (p-14-3-3 sigma) and expressing MDM2 (p-MDM2), as well as the short-hairpin (sh) RNA directed against 14-3-3 sigma (sh-14-3-3 sigma) and its corresponding negative control (sh-NC) were designed and synthesized by GenePharma (Shanghai, China). For comparison, the empty pcDNA3.1 vector was applied for the negative control. Human breast cells were transfected at 50% confluency using Lipofectamine 3000 (Life Technologies, Carlsbad, MA, USA) following manufacturer’s instructions and harvested for subsequent experiments at 48 h after transfection.

### 2.3. Cell-Counting Kit 8 (CCK8) Assay

Human breast cells were inoculated into a 96-well plate (5000 cells per well) and maintained for 24 h, 48 h and 72 h in the incubator at 37 °C. Cell-counting kit 8 (CCK8) assay was conducted following manufacturer’s instructions. All conditions were repeated in 6 wells. For each well, 90 μL of medium and 10 μL of CCK8 (WST-8; ab228554; Abcam, Cambridge, UK) were added. The plate was then protected from the light and incubated for another 2 h. Finally, the optical density (OD) value of the culture at 460 nm was obtained using a microplate reader.

### 2.4. Clone-Formation Assay

Cell clone-formation ability was evaluated by plate clone-formation assay as previously described [11]. Human breast cells were resuspended at a density of 2000 cells/mL and seeded 100 μL in each well of 6-well plates. The cells were cultured for two weeks with continuous observation for cloning visibility. The culture medium was refreshed every two days. When most wells achieved no fewer than 50 clones, the breast cells were subsequently covered with 4% paraformaldehyde solution for 15 min, followed by adding moderate Giemsa to stain for 30 min. Pictures were taken and the number of clones that were more than 10 cells was calculated using a microscope.

### 2.5. Cell Apoptosis Assay

To monitor the incidence of apoptosis, cell apoptosis assay was conducted by using a ReadiDrop™ propidium iodide (PI) and fluorescein isothiocynate-conjugated Annexin V staining kit following manufacturer’s instructions (Bio-Rad, Hercules, CA, US). The cells were seeded at a density of 2 × 10^6^ cells in a 6-well plate, and when the cells reached 80% confluence they were treated with ZLM-7 or vehicle control for 8 h. After 24 h the cells were washed with PBS, collected and then suspended in 500 μL flow-cytometry buffer (1× phosphate-buffered saline with 3% BSA) according to the manufacturer’s instructions. Annexin V and PI were added to the sample. Samples were then incubated for 30 min at 37 °C. The change in apoptosis was analyzed by measuring the fluorescence using a Flow Cytometer (Beckman Coulter, Brea, CA, USA) after staining.

### 2.6. Cell-Cycle Analysis

Cell-cycle analysis was conducted by Propidium Iodide Flow Cytometry Kit (ab139418, Abcam, Cambridge, UK) following manufactures’ instructions. Human breast cells were collected and washed with PBS. The cells were then fixed with ice-cold 70% ethanol at 4 °C for 30 min. The cells were washed with PBS twice after ethanol removal and spined out of ethanol in a centrifuge. Cells were then incubated with 100 µg/mL RNase at 37 °C for 15 min to ensure only DNA was stained. After staining with 50 µg/mL PI for 30 min in the dark, cell-cycle distribution was evaluated by analyzing the data capture by a Flow Cytometer (Beckman Coulter, Brea, CA, USA).

### 2.7. Xenograft Animal Model

Ethical approval was obtained from Medical Ethics Committee of Xiangya Hospital, Central South University. All procedures in animal experiments were carried out in accordance with the guide for the care and use of laboratory animals. BALB/c nude mice at approximately seven weeks’ old were obtained from SJA Laboratory Animal Co., Ltd. (Hunan, China). All animals were housed under standard condition with a 12 h light/dark cycle at 21 °C and a humidity of 55%. Xenografting is accomplished by subcutaneous administration of breast cancer cells or cells transfected with either sh-14-3-3 sigma or sh-NC (5 × 10^6^ cells per animal) into the back of the animal adjacent to the left front limb as described previously [25]. ZLM-7 treatment was given by intraperitoneal injection at a dose of 15 mg/kg/day based on previous reports [10]. Tumor size was evaluated and recorded every week and tumor volume was calculated simultaneously. The duration of the experiment was 28 days. At the end of the study, all animals were anesthetized. Meanwhile, tumors were harvested without the surrounding tissues. The weight of the tumor was recorded.

### 2.8. Quantitative Real-Time Polymerase Chain Reaction (qPCR)

RNA samples were isolated and purified from human breast cells and mouse breast cancer tissues using TRIzol reagent (Invitrogen, Carlsbad, CA, USA) and RNA purification kit. cDNA was synthesized from RNA samples using a HiFiScript cDNA synthesis kit following per manufacturer’s instructions (Life Technologies, Carlsbad, MA, USA). Subsequently, quantitative real-time PCR was conveyed by using an UltraSYBR Mixture kit (Thermo Fisher, Waltham, MA, USA) in a 96-well plate on ABI Prism 7500 system (Applied BioSystems, Waltham, MA, USA). The relative fold change of expression for each gene was calculated using 2^−∆∆Ct^ method [26]. The primers used in the qPCR were as follows: 14-3-3 sigma: forward 5′-GGA TCC CAC TCT TCT TGC AG-3′ and reverse 5′-CTG TCC AGT TCT CAG CCA CA-3′; MDM2: forward 5′-TGT TTG GCG TGC CAA GCT TCT GA-3′ and reverse 5′-GAC AGA TGT ACC TGA GTC CGA TG-3′. GAPDH: forward 5′-CCA GCT CCT GTC ATC TGA-3′ and reverse 5′-GAT GTA GAC AGA TCG TAG T-3′. The housekeeping gene GAPDH served as an internal control for comparisons of gene-expression data.

### 2.9. Western Blotting Assay

Total proteins used in Western blotting experimentation were obtained from human breast cells or tumor-tissue samples by using radio-immunoprecipitation assay buffer (Thermo Fisher, Waltham, MA, USA) containing a protease inhibitor cocktail. Protein concentrations were analyzed by a Direct Detect Spectrometer (Merck, Darmstadt, Germany). An amount of 20 ug total protein was electrophoresed at reducing conditions. Separated proteins after gel electrophoresis were then transblotted onto polyvinylidene difluoride (PVDF) membranes. The membranes were then blocked with 5% nonfat milk blocking buffer at room temperature for 1 h to prevent nonspecific binding, followed by incubation with a series of primary antibodies against 14-3-3 sigma (ab193667, dilution 1:1000), MDM2 (ab259265, dilution 1:1000), P21 (ab109520, dilution 1:2000), cyclin D1 (ab16663, dilution 1:200), CDK2 (ab32147, dilution 1:5000), CDK6 (ab124821, dilution 1:50,000) and GAPDH (ab179467, dilution 1:5000) overnight at 4 °C. The antibodies were bought from Abcam (Cambridge, UK). After washing with TBST buffer (20 mM Tris, 137 mM NaCl, 0.1% Tween-20, pH 8.0) 3 times, membranes were incubated with horseradish peroxidase-conjugated secondary antibodies in blocking buffer at room temperature for 2 h. Finally, the immunoreactive bands on blot were visualized and detected by a BioRad Chemidoc MP system. The fold changes of the proteins of interest were normalized to that of GAPDH.

### 2.10. Statistical Analysis

All data were analyzed using IBM SPSS Statistics 23 (IBM, Armonk, NY, USA). Comparisons between groups were performed by using Student t-test or one-way analysis of variance (ANOVA) as appropriately. A value of *p* < 0.05 was accepted as statistically significant.

## 3. Results

### 3.1. ZLM-7 Upregulated 14-3-3 Sigma Expression but Downregulated MDM2 Expression in Breast Cancer Cells

**Figure 1:**
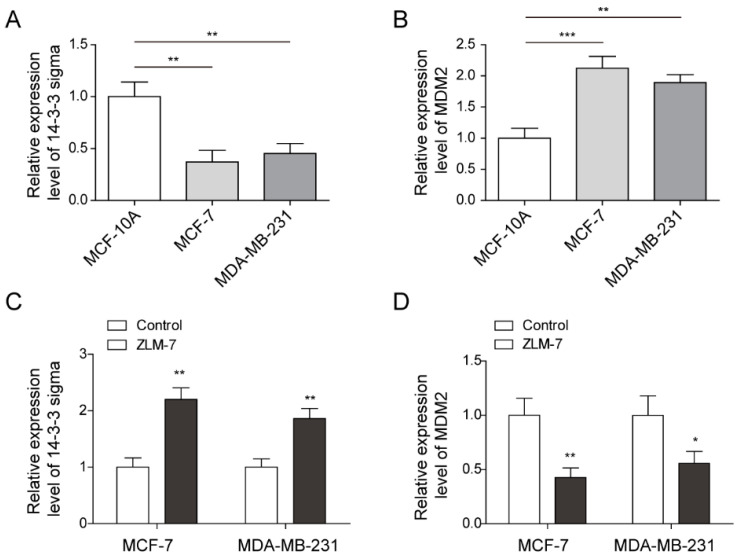
ZLM-7 upregulated 14-3-3 sigma expression but downregulated MDM2 expression in breast cancer cells. (**A**,**B**). Relative gene expressions of 14-3-3 sigma and MDM2 in MCF-10A, MCF-7 and MDA-MB-231 cells measured by qPCR. (**C**,**D**). Relative gene expression of 14-3-3 sigma and MDM2 with or without ZLM-7 treatment. Gene expressions were measured by qPCR. * *p* < 0.05, ** *p* < 0.01, *** *p* < 0.001.

As described previously, 14-3-3 sigma and MDM2 are associated with the oncogenesis in breast cancer. In order to evaluate the action of ZLM-7 on the expressions of 14-3-3 sigma and MDM2 in breast cancer, we firstly investigated the expressions of those genes in two independent human breast cancer cell lines, MDA-MB-231 and MCF-7, as well as in the normal MCF-10A mammary epithelial cell line. The qPCR analysis was conveyed for gene expression and the result revealed that the 14-3-3 sigma expression in both breast cancer cells was decreased while the MDM2 expression was increased in comparison with those in normal breast epithelial cells (Figure 1A,B). Then, we further examined the action of ZLM-7 on those gene expressions in those breast cell lines. The result showed that ZLM-7 treatment could alter the expression of 14-3-3 sigma and MDM2 by upregulating the expression of 14-3-3 sigma, and interestingly, downregulating MDM2 expression in breast cancer cells (Figure 1C,D). These results suggest a possible regulatory action of ZLM-7 on 14-3-3 sigma and MDM2 in breast cancer.

### 3.2. ZLM-7 Inhibited Cell Proliferation and Promoted Apoptosis by Upregulating 14-3-3 Sigma in Breast Cancer Cells

**Figure 2:**
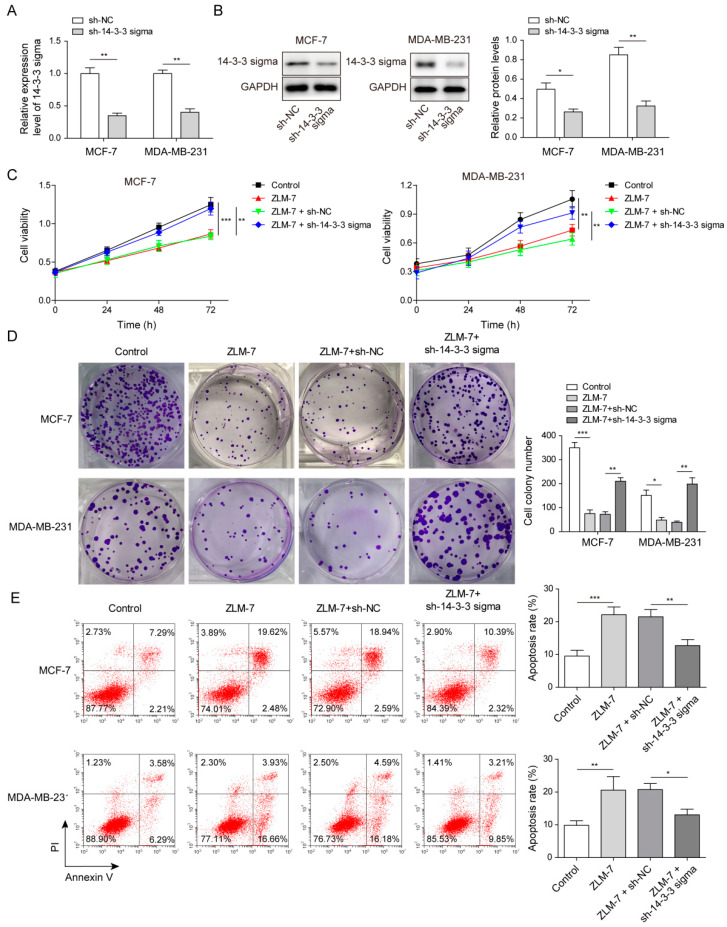
ZLM-7 suppressed cell proliferation and promoted apoptosis by upregulating 14-3-3 sigma in breast cancer cells. (**A**) Relative gene expression of 14-3-3 sigma in MCF-7 and MDA-MB-231 cells transfected with either sh-NC or sh-14-3-3 sigma measured by qPCR. (**B**) Relative protein level of 14-3-3 sigma in MCF-7 and MDA-MB-231 cells transfected with either sh-NC or sh-14-3-3 sigma measured by Western blotting. (**C**) Cell viability at 24 h, 48 h and 72 h measured by CCK8 assay. (**D**) Cell proliferation ability assessed by clone-formation assay in MCF-7 and MDA-MB-231 cells transfected with either sh-NC or sh-14-3-3 sigma. (**E**) Cell apoptosis analyzed by flow cytometry. * *p* < 0.05, ** *p* < 0.01, *** *p* < 0.001.

We then explored the effect of ZLM-7 induced 14-3-3 sigma upregulation on proliferation and apoptosis in those breast cancer cell lines by specific knockdown of genes of interest. Cells were firstly transfected with sh-14-3-3 sigma to knockdown the 14-3-3 sigma gene. The results of qPCR and Western blot confirmed that transfection with sh-14-3-3 sigma successfully repressed the expression of 14-3-3 sigma, when compared with transfection with sh-NC (Figure 2A,B). We then applied ZLM-7 treatment on those transfected cells. ZLM-7 treatment suppressed cell proliferation in a time-dependent fashion while silencing of 14-3-3 sigma abolished the inhibitory effect of ZLM-7 (Figure 2C). Similarly, ZLM-7 treatment reduced the clone formation of breast cancer cells, which was reversed by 14-3-3 sigma silencing (Figure 2D). After ZLM-7 treatment, the apoptosis of breast cancer cell was increased while 14-3-3 sigma silencing reversed the action of ZLM-7 on apoptosis (Figure 2E). We therefore deduced from those results that ZLM-7 suppressed proliferation and promoted apoptosis by upregulating 14-3-3 sigma expression in breast cancer cells.

### 3.3. ZLM-7 Blocked Cell-Cycle Progression via Upregulating 14-3-3 Sigma

**Figure 3:**
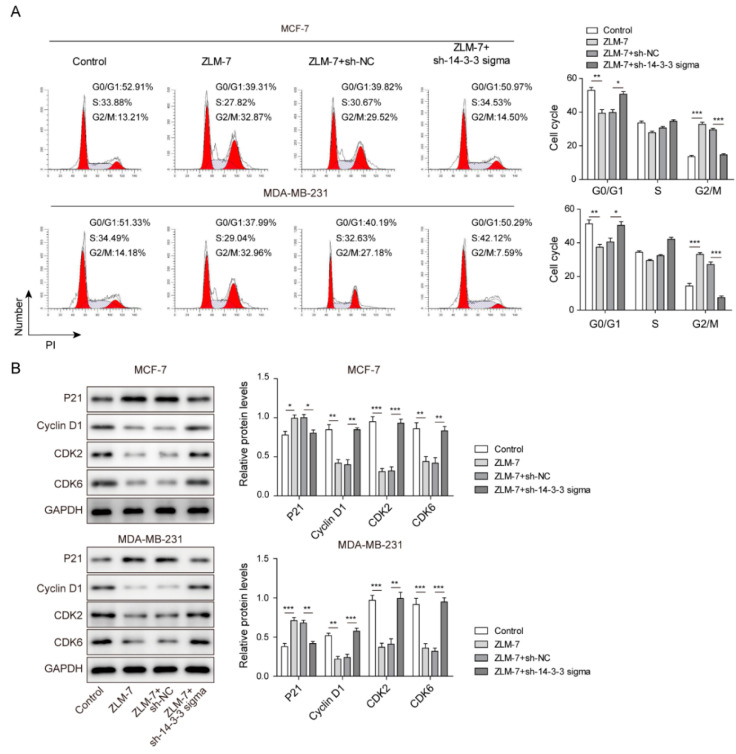
ZLM-7 blocked cell-cycle progression via upregulating 14-3-3 sigma. (**A**) Cell-cycle analyzed via flow cytometry. (**B**) Relative protein levels of cell-cycle regulatory proteins measured by Western blotting. MCF-7 and MDA-MB-231 cells transfected with either sh-NC or sh-14-3-3 sigma were treated with ZLM-7 for 8 h. * *p* < 0.05, ** *p* < 0.01, *** *p* < 0.001.

We further studied the action of ZLM-7 on cell-cycle progression and the possible regulating mechanisms. Our data demonstrated that ZLM-7 treatment produced a significant accumulation of cells in the G2/M phase of the cell cycle; meanwhile, there was a substantial decrease in the percentage of cells in the G0/G1 phase (Figure 3A). Based on those findings, we further studied the proteins associated with cell-cycle progression. The protein expressions of cell-cycle regulatory proteins were measured by Western blot assay. In agreement with the results from the cell-cycle analysis, we found that the protein expression of the negative cell-cycle regulator P21 was markedly increased in breast cancer cells after ZLM-7 treatment, which was accompanied with decreased expressions of cyclin D1, CDK2 and CDK6 (Figure 3B). Notedly, transfection with sh-14-3-3 sigma abolished those effects of ZLM-7 on cell-cycle progression and distribution in addition to the expressions of those cell-cycle regulatory proteins in breast cancer cells (Figure 3A,B). Taken together, ZLM-7 could induce cell-cycle arrest via upregulating 14-3-3 sigma.

### 3.4. 14-3-3 Sigma Could Negatively Regulate the Expression of MDM2 in Breast Cancer Cells

**Figure 4:**
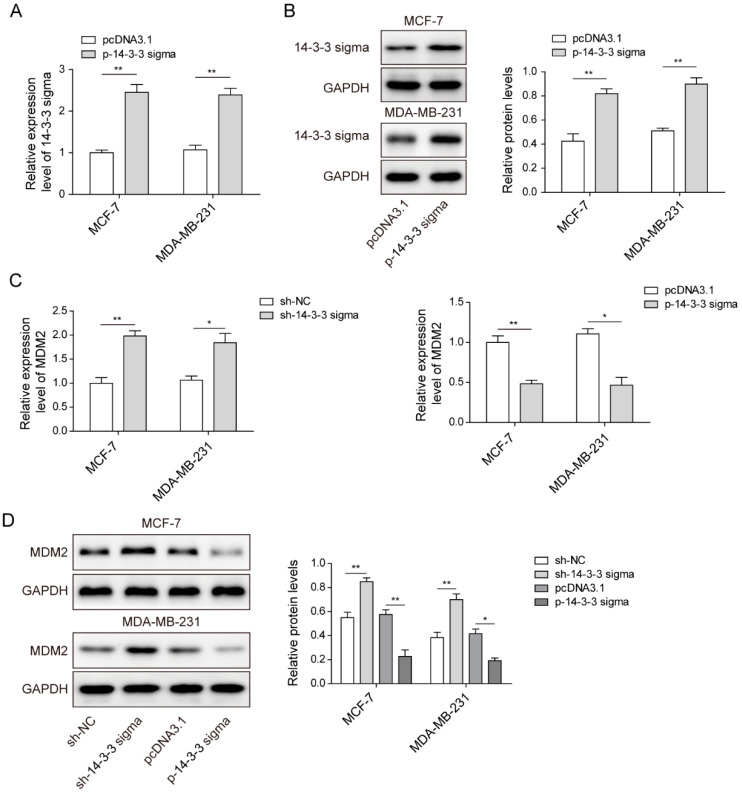
14-3-3 sigma could negatively regulate the expression of MDM2 in breast cancer cells. (**A**) Relative gene expression of 14-3-3 sigma in MCF-7 and MDA-MB-231 cells transfected with either pcDNA3.1 or p-14-3-3 sigma measured by qPCR. (**B**) Relative protein level of 14-3-3 sigma in MCF-7 and MDA-MB-231 cells transfected with either pcDNA3.1 or p-14-3-3 sigma measured by Western blotting. (**C**) Relative gene expression of MDM2 in MCF-7 and MDA-MB-231 cells transfected with either pcDNA3.1 or p-14-3-3 sigma measured by qPCR. (**D**) Relative protein level of MDM2 in MCF-7 and MDA-MB-231 cells transfected with either pcDNA3.1 or p-14-3-3 sigma measured by Western blotting. * *p* < 0.05, ** *p* < 0.01.

We then pursued to reveal the downstream target of 14-3-3 sigma and the potential regulations. Human breast cancer cells were transfected with p-14-3-3 sigma to overexpress the 14-3-3 sigma gene. qPCR and Western blotting were then carried out to confirm the overexpression of 14-3-3 sigma in RNA and protein levels after cell transfection (Figure 4A,B). By using the successfully established 14-3-3 sigma knockdown cells and the 14-3-3 sigma overexpression cells, we next explored the potential regulatory relationship between 14-3-3 sigma and MDM2 in breast cancer. Interestingly, we found that the gene and protein expressions of MDM2 were significantly increased in 14-3-3 sigma knockdown cells, while the MDM2 expression was decreased in cells overexpressed 14-3-3 sigma (Figure 4C,D), confirming the regulatory role of 14-3-3 sigma in MDM2 expression.

### 3.5. Overexpressed 14-3-3 Sigma Inhibited Cell Proliferation and Facilitated Apoptosis by Reducing MDM2 Expression in Breast Cancer Cells

**Figure 5:**
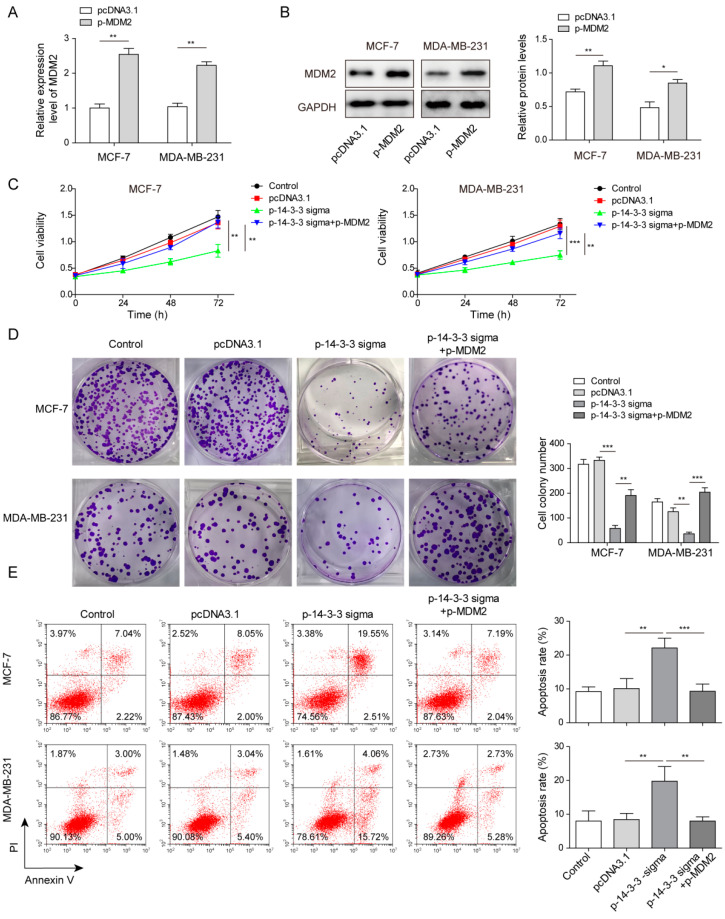
Overexpressed 14-3-3 sigma inhibited cell proliferation and promoted apoptosis by reducing MDM2 expression in breast cancer cells. (**A**) Relative gene expression of MDM2 in MCF-7 and MDA-MB-231 cells transfected with either pcDNA3.1 or p-MDM2 measured by qPCR. (**B**) Relative protein level of MDM2 in MCF-7 and MDA-MB-231 cells transfected with either pcDNA3.1 or p-MDM2 measured by Western blot. (**C**) Cell viability at 24 h, 48 h and 72 h measured by CCK8 assay. (**D**) Cell proliferation ability assessed by clone formation assay in MCF-7 and MDA-MB-231 cells transfected with pcDNA3.1 or p-14-3-3 sigma or co-transfected with p-14-3-3 sigma and p-MDM2. (**E**) Cell apoptosis analyzed by flow cytometry. * *p* < 0.05, ** *p* < 0.01, *** *p* < 0.001.

We further analyzed the function of MDM2 in breast cancer cells, in terms of cell proliferation and apoptosis. Human breast cancer cells were transfected with p-MDM2 and the success of transfection was validated by qPCR and Western blotting assay, where increased gene and protein expressions were observed in transfected cells (Figure 5A,B). By using qPCR and Western blotting, we also found that overexpressed MDM2 could attenuate the inhibition of cell proliferation and clone formation induced by 14-3-3 sigma overexpression (Figure 5C,D). Similarly, MDM2 overexpression antagonized the apoptosis-promoting effect of 14-3-3 sigma overexpression in the transfected breast cancer cells (Figure 5E). Those findings implicit that 14-3-3 sigma regulates cell proliferation and apoptosis via mediating MDM2.

### 3.6. 14-3-3 Sigma Blocked Cell-Cycle Progression by Suppressing MDM2 Expression in Breast Cancer Cells

**Figure 6:**
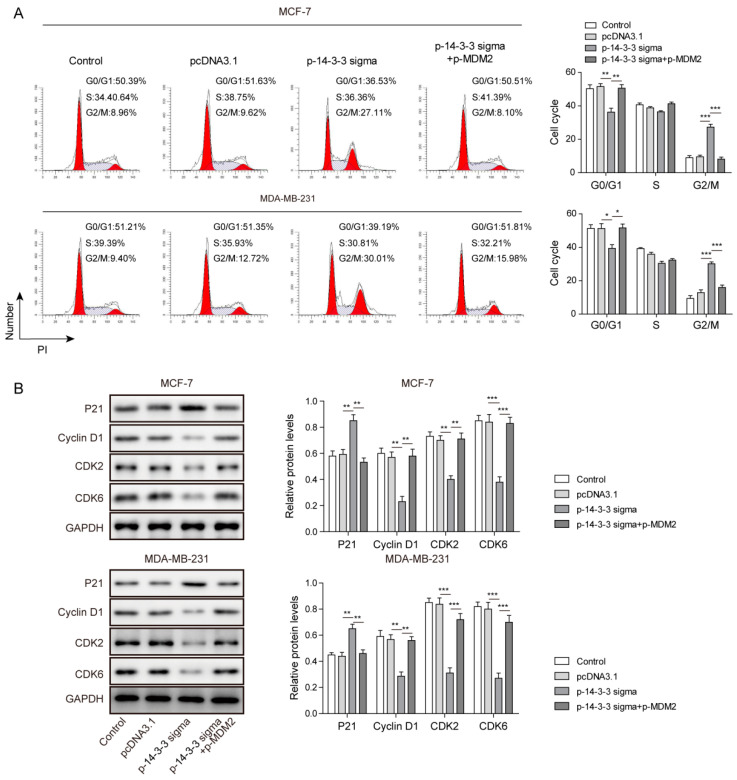
14-3-3 sigma blocked cell-cycle progression by suppressing MDM2 expression in breast cancer cells. (**A**) Cell-cycle analyses via flow cytometry. (**B**) Relative protein levels of cell-cycle regulatory proteins measured by Western blot. MCF-7 and MDA-MB-231 cells transfected with pcDNA3.1 or p-14-3-3 sigma or co-transfected with p-14-3-3 sigma and p-MDM2. * *p* < 0.05, ** *p* < 0.01, *** *p* < 0.001.

We then investigated the action of MDM2 overexpression on cell-cycle progression. The results from flow cytometry revealed that 14-3-3 sigma overexpression markedly raised the percentage of cells in the G2/M phase and diminished cells in the G0/G1 phase, and therefore caused G2/M phase arrest. MDM2 overexpression could reverse those effects induced by 14-3-3 sigma (Figure 6A). When 14-3-3 sigma was overexpressed, Western blot assay presented increased protein expression of P21 and decreased levels of cyclin D1, CDK2 and CDK6 in breast cancer cells. However, those effects were abrogated by the overexpression of MDM2 (Figure 6B). Those findings from cell-cycle analysis and Western blotting suggest that 14-3-3 sigma blocks cell-cycle progression by suppressing MDM2.

### 3.7. ZLM-7 Inhibited Tumor Growth via Regulating 14-3-3 Sigma/MDM2 Axis In Vivo

**Figure 7:**
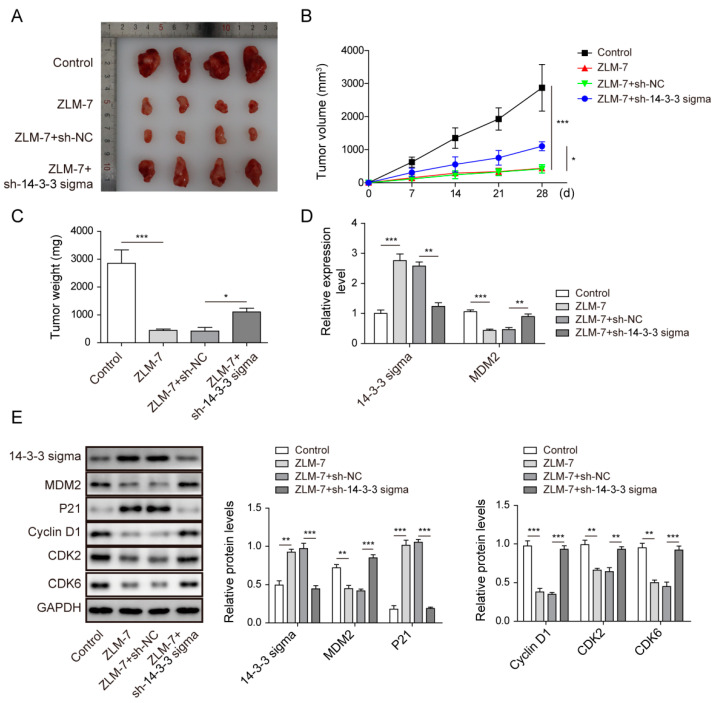
ZLM-7 suppressed tumor growth via regulating 14-3-3 sigma/MDM2 axis in vivo. Mice were inoculated subcutaneously with breast cancer cells or cells transfected with either sh-14-3-3 sigma or sh-NC. (**A**) Xenografts harvested from the animals; (**B**,**C**) Tumor volume and weight with ZLM-7 treatment for 28 days. (**D**) Relative gene expressions of 14-3-3 sigma and MDM2 in xenografts measured by qPCR. (**E**) Relative protein levels of 14-3-3 sigma, MDM2 and cell-cycle regulatory proteins in xenografts detected by Western blotting. * *p* < 0.05, ** *p* < 0.01, *** *p* < 0.001.

To clarify the regulatory role of ZLM-7 in the 14-3-3 sigma/MDM2 axis, we examined the action of ZLM-7 treatment in a breast cancer xenograft mouse model. ZLM-7 treatment significantly delayed tumor growth, in terms of tumor volume and weight, while knockdown of 14-3-3 sigma partial reversed the action of ZLM-7 (Figure 7A–C). Consistent with the observations from in vitro experiments, ZLM-7 could upregulate the expression of 14-3-3 sigma while downregulating the expression of MDM2 in xenograft mice, whereas those effects of ZLM-7 were reversed by 14-3-3 sigma knockdown (Figure 7D). We further explored the action of ZLM-7 on the cell cycle by measuring the changes of the cell-cycle regulatory proteins in tumors. As expected, ZLM-7 increased the protein levels of 14-3-3 sigma and p21 and decreased levels of MDM2, cyclin D1, CDK2 and CDK6. Finally, those changes induced by ZLM-7 were abolished by 14-3-3 sigma silencing (Figure 7E). In sum, ZLM-7 could inhibit MDM2 expression via 14-3-3 sigma and thus inhibited tumor growth in vivo.

## 4. Discussion

In recent years, breast cancer has surpassed lung cancer to be the most frequently diagnosed cancer and is becoming the leading global cause of cancer-related death in females [27]. Although great advances have been accompanied by significant development in the clinical diagnosis and management of breast cancer, the prognosis is still undesirable. In present study, we revealed that ZLM-7 could block cell-cycle progression and therefore inhibit tumor growth and progression of breast cancer, depending on the regulation of the 14-3-3 sigma/MDM2 axis.

Due to the limitation of CA-4, a great number of novel CA-4 derivatives in different structures have been developed and have demonstrated more potent antimitotic activities than CA-4 [4]. The published research on ZLM-7 was very limited, but the antineoplastic and antiangiogenic actions of ZLM had been highlighted by two previous studies. In the study conducted by Su et al., ZLM-7 was found to be a potent angiogenesis inhibitor by blocking vascular endothelial growth factor (VEGFR) signaling, where ZLM-7 possessed comparable antiangiogenic and antitumor functions as CA-4, but had lower toxicity [10]. In another study, Li et al. observed consistent results that ZLM-7 suppressed the development, growth and new blood-vessel formation of breast cancer via decreasing VEGFA expression [11]. In agreement with those studies, we found that ZLM-7 could block cell-cycle progress and inhibit tumor growth in breast cancer xenografts. Notedly, a novel mechanism had been revealed that ZLM-7 suppressed MDM2 by upregulating 14-3-3 sigma expression.

As a pivotal mediator of p53, 14-3-3 sigma controls the entry into mitosis responding to DNA damage and inhibits MDM2-mediated p53 ubiquitination during the tumorigenesis of breast cancer and other tumor types [14,28]. Indeed, breast cancer cells with BRCA1 deficiency caused downregulation of 14-3-3 sigma and could not hinder cell-cycle progression towards the G2/M phase [29]. It was also reported that deficiency of 14-3-3 sigma function resulted from hypermethylation of its promoter is a prevention and early event in the oncogenesis in the breast [30,31]. Therefore, targeting 14-3-3 sigma to enhance or restore its function on p53 was considered to be a promising treatment for cancers [32]. Moreover, several studies have implicated that 14-3-3 sigma could be a potential and promising candidate for tumor therapy [13]. For example, small molecule stabilizers had been designed to enhance the binding capacity of 14-3-3 sigma to estrogen receptor α (Erα), which is a potent driver of tumor cell growth in breast cancer, resulting in decreased transcriptional activity of ERα [33,34]. 14-3-3 sigma has also been demonstrated to prevent tumor-promoting metabolic processes, such as glutaminolysis, glycolysis and mitochondrial function, by boosting protein breakdown of the carcinogenic factor c-Myc [35].

MDM2 has been found to exert suppressive effects on p53 via binding p53 and shuttling the p53 protein to proteasome and subsequently being degraded [36,37]. Moreover, it is also reported that overexpression of MDM2 could attenuate the activities of p53 to promote cell-cycle arrest and cell apoptosis [38]. In addition, accumulating evident suggested that MDM2 could retain its action in cell-cycle regulation, cell differentiation, apoptosis and other processes in the absence of p53 [39]. In addition, the CA-4 analog SQ induced p53-independent apoptosis by inhibiting MDM2 [20]. This evidence suggests that MDM2 is a valuable target in cancer treatment, regardless of the p53 status. In our study, we found that ZLM-7 treatment was able to upregulate 14-3-3 sigma expression and inhibit MDM2 expression and subsequently lead to apoptosis and cell-cycle arrest, suggesting the possible extensive use of ZLM-7 for cancer therapy. Previous studies have found that ZLM-7 regulated the angiogenesis of breast cancer cells [10,11]. Angiogenesis is an important process of tumorigenesis [40]. After the angiogenesis switch is turned on, the paracrine effect during tumor growth causes rapid tumor growth. This paracrine effect is caused by growth factors, and the loss of the supporting effect of these growth factors on tumor cells will induce tumor cell apoptosis [41]. Among them, vascular endothelial growth factor (VEGF) is the most important [42]. It was reported that inhibition of VEGF could induce apoptosis of breast cancer cells [43]. In breast cancer, whether ZLM-7 promotes tumor cell apoptosis by boosting angiogenesis medicated by VEGF remains to be further explored. The present study examined the mechanism of ZLM-7 on cell proliferation in breast cancer cells. However, further studies are warranted to determine the major contributor of the observed antitumor effect of ZLM-7 in vivo, either the regulation of proliferation or angiogenesis.

## 5. Conclusions

The present study suggests that the treatment of ZLM-7 could block cell-cycle progression and induce cell-cycle arrest of breast cancer both in vitro and in vivo via regulating 14-3-3 sigma/MDM2 axis. Our findings would provide a better understanding and novel insights of the regulatory machinery of cell-cycle progression in breast cancer and propose further evidence for the therapeutic use of ZLM-7.

## Data Availability

Data is contained within the article.
